# FBXO11 Mediates Ubiquitination of ZEB1 and Modulates Epithelial-to-Mesenchymal Transition in Lung Cancer Cells

**DOI:** 10.3390/cancers16193269

**Published:** 2024-09-26

**Authors:** Xinyue Zhao, Zhihui Han, Ruiying Liu, Zehao Li, Ling Mei, Yue Jin

**Affiliations:** 1 Edmond H. Fischer Signal Transduction Laboratory, School of Life Sciences, Jilin University, Changchun 130012, China; zhaoxinyue20@mails.jlu.edu.cn (X.Z.); hanzhihui962024@163.com (Z.H.); liury22@mails.jlu.edu.cn (R.L.); zehao21@mails.jlu.edu.cn (Z.L.); meiling23@mails.jlu.edu.cn (L.M.); 2 National Engineering Laboratory of AIDS Vaccine, School of Life Sciences, Jilin University, Changchun 130012, China; 3 Key Laboratory of Organ Regeneration & Transplantation of the Ministry of Education, Jilin University, Changchun 130061, China

**Keywords:** ZEB1, FBXO11, lung cancer, EMT, invasion and metastasis

## Abstract

**Simple Summary:**

Epithelial-to-mesenchymal transition (EMT) plays a critical role in cancer progression, contributing to the invasive and migratory abilities of tumor cells. In this study, we show that FBXO11 promotes the degradation of ZEB1, a key EMT regulator, via ubiquitination. Loss of FBXO11 increases ZEB1 levels, enhancing the invasiveness of lung cancer cells, while its overexpression reduces ZEB1 and suppresses invasion. Importantly, higher FBXO11 expression is associated with better prognosis in non-small cell lung cancer (NSCLC), highlighting its potential role as a therapeutic target for controlling EMT and cancer metastasis.

**Abstract:**

Epithelial-to-mesenchymal transition (EMT) affects the invasion and migration of cancer cells. Here, we show that FBXO11 recognizes and promotes ubiquitin-mediated degradation of ZEB1. There is a strong association between FBXO11 and ZEB1 in non-small cell lung cancer (NSLC) in a clinical database. FBXO11 interacts with ZEB1, a core inducer of EMT. FBXO11 leads to increased ubiquitination and proteasomal degradation of ZEB1. Depletion of endogenous FBXO11 causes ZEB1 protein accumulation and EMT in A549 and H1299 cells, while overexpression of FBXO11 reduces ZEB1 protein abundance and cellular invasiveness. Importantly, the depletion of ZEB1 suppresses the increased migration and invasion of A549 and H1299 cells promoted by the depletion of FBXO11. The same results are shown in xenograft tumors. High FBXO11 expression is associated with a favorable prognosis in NSLC. Collectively, our study demonstrates that FBXO11 modulates EMT by mediating the stability of ZEB1 in lung cancer cells.

## 1. Introduction

According to GLOBOCAN’s Global Cancer Statistics 2023, lung cancer is the number-one cause of death [1,2]. There are two main histological types of lung cancer, namely small cell lung cancer (SCLC) and non-small cell lung cancer (NSCLC). Of these, NSCLC accounts for about 85% [3,4,5,6]. Metastasis is the primary cause of death in patients with lung cancer [7]. Tumor cell invasion and metastasis are closely linked to epithelial-to-mesenchymal transition (EMT). EMT transforms polarized epithelial cells, typically anchored to the basement membrane, into mesenchymal cells with increased migration, invasiveness, and resistance to apoptosis [8]. ZEB1, also known as TCF8 or δEF1, is a key transcriptional regulator of EMT [9,10]. ZEB1 transcriptionally represses the expression of E-cadherin by binding to its promoter region [11]. Research shows that ZEB1 can induce EMT in breast cancer [10], osteosarcoma [12], lung cancer [13], melanoma [14], and other epithelioma, leading to tumor metastasis and promoting drug resistance. High expression of ZEB1 is related to the poor prognosis of cancer patients. Studies by Manshouri R et al. have shown that ZEB1 can become a therapeutic target for metastatic NSCLC. Various F-box proteins, including TrCP1/Fbxw1, Fbxw7, Ppa/Fbxl14, Fbxl5, Fbxo11, and Fbxo45, have been implicated in EMT by facilitating the degradation of EMT-related transcription factors (EMT-TFs) [15,16,17,18,19,20]. FBXO11, as a member of the SCF (Skp1-Cul1-F-box) ubiquitin ligase complex, exhibits E3 ubiquitin ligase activity and methyltransferase activity [21]. FBXO11 was first mentioned as a ubiquitin ligase in diffuse large B-cell lymphomas (DLBCLs), targeting BCL6 for its degradation and stabilization and, thus, inhibiting cell proliferation and inducing cell death [22]. FBXO11 can promote CDT2 polyubiquitylation, and degradation controls the timing of cell-cycle exit [23,24]. FBXO11 can regulate the invasive metastasis of tumor cells by associating with the EMT-related factor Snail, as well as the classical PI3k/Akt signaling pathway [25,26]. Recently, FBXO11 has been reported to be a major negative regulator of MHC class II through ubiquitin-dependent proteasomal degradation of CIITA in breast cancer [27]. In hepatocellular carcinoma, FBXO11 mediates heterogeneous ribonucleoprotein A2/B1 ubiquitination to regulate lipid metabolic reprogramming and promote tumorigenesis [28]. The modulation of cisplatin resistance by the miR-324-5pFBXO11 axis is observed in lung cancer [29]. In this study, we found that FBXO11 interacts with the ZEB1 CZF domain and promotes it ubiquitination and degradation. FBXO11 activity affects the stability and function of the ZEB1 protein, resulting in marked biological effects in vivo. This molecular interplay shapes the proliferative and metastatic competencies of lung cancer cells, thereby transforming their dissemination behavior within the biological milieu.

## 2. Materials and Methods

### 2.1. Cell Culture and Transfection

Human embryonic kidney cell HEK293T and human lung adenocarcinoma cell line A549 cells were cultured in DMEM (Gibco, Waltham, MA, USA, C11995500B) with 10% FBS (Kangyuan Biotechnology, Shanghai, China, KY-01000). Human lung adenocarcinoma cell line H1299 cells were grown in RPMI-1640 (Gibco) with 10% FBS. Transfections were performed using Lipofectamine 2000 (Invitrogen, Carlsbad, CA, USA) per the manufacturer’s instructions.

### 2.2. Plasmids and Mutagenesis

ZEB1 and FBXO11 cDNA were synthesized and cloned into lentiviral vector pLVX-IRES-neo and pET28a by Miaoling Biology (Wuhan, China). FBXO11 was cloned into a pLVX-IRES-neo vector with Myc tagged. ZEB1 was cloned into a pLVX-IRES-neo vector with Flag tagged. FBXO11 and ZEB1 full-length and truncated mutants of ZEB1 were cloned into a pET28a vector.

### 2.3. Western Blotting and Immunoprecipitation

Western blotting was conducted as previously described [30]. Cell extracts were prepared with cold RIPA buffer (150 mM NaCl, 50 mM Tris pH 7.5, 1% NP-40, and 10% glycerol). Proteins were separated on 6–12% gels and transferred to PVDF membranes. Membranes (Millipore, Shanghai, China, IPVH00010) were probed with primary antibodies, including FBXO11 (Proteintech, Wuhan, China, #67365-1-Ig), ZEB1 (Santa Cruz Biotechnology, Oregon, USA, #515797), E-cadherin (Proteintech, #20874-1-AP), N-cadherin (BD Transduction Laboratories, #610920), GFP (Proteintech, #66002-1-Ig), and GAPDH (GAPDH; Bioss, Woburn, MA, USA, #0978M).

For immunoprecipitation assays, indicated plasmids were co-transfected with HEK293T cells in six-well plates. After 18 h, transfected cells were treated with 10 μM proteasome inhibitor MG132 (Sigma-Aldrich, St. Louis, MO, USA, C2211) for 6 h and lysed with cold RIPA lysate buffer to collect the supernatant and retain the input. The supernatants were combined with RIPA binding buffer (150 mM NaCl, 50 mM Tris PH 7.5, and 10% glycerol) and 20 μL protein A/G agarose beads (Invitrogen, 20421) and incubated at 4 °C for 6 h. The mixture was subjected to immunoprecipitation with either Flag (Proteintech, #66008-4-Ig) or Myc (Proteintech, #60003-2-Ig) antibodies and slowly overturned overnight at 4 °C. The agarose beads–antigen–antibody mixture was washed thoroughly with RIPA buffer, and Western blot analysis was conducted with indicated antibodies.

### 2.4. His-Tagged Protein Interaction Pull-Down Assay

FBXO11 wild-type or ZEB1 truncated cDNA was amplified by PCR and cloned into bacterial expression vector pET28a with an N-terminal His tag. The plasmids were transformed into BL21(DE3) (TransGen, Beijing, China, CD601-02). His-tagged FBXO11 protein or ZEB1 truncated proteins were induced by 0.4 mM IPTG at 21 °C for 16 h and purified by Ni-NTA beads (Qiagen, Hilden, Germany, 1018244). The His-tagged FBXO11 proteins or ZEB1-truncated proteins that bound to Ni-NTA beads were washed with PBS and collected at 4 °C. HEK293T cells transfected with ZEB1-Flag or FBXO11-Myc expression constructs were harvested in lysis buffer (50 mM NaH_2_PO_4_.H_2_O pH 8, 300 mM NaCl, 10 mM imidazole, protease inhibitor cocktail) and sonicated (power, 300 W; time, 10 s; interval, 50 s). The sonicated cell lysates were incubated with purified sFBXO11 or ZEB1-truncated proteins and Ni-NTA beads. Proteins that bound to Ni-NTA beads were eluted (50 mM NaH_2_PO_4_.H_2_O, pH 8, 300 mM NaCl, 20 mM imidazole, protease inhibitor cocktail) and subjected to Western blot analysis with indicated antibodies.

### 2.5. Immunofluorescence

An immunofluorescence assay was performed as previously described [31]. Fluorescence photography was captured with a laser scanning confocal microscope (Leica Microsystems, Wetzlar, Germany, LSM 710). Primary antibodies included FBXO11 (Novussbio, Centennial, CO, USA 100-59826), ZEB1 (Santa Cruz, Dallas, TX, USA, #515797), and E-cadherin (Proteintech, 20874-1-AP). Secondary antibodies included CoraLite488-conjugated (Proteintech, SA00013-2), CoraLite594-conjugated (Proteintech, SA00013-3), and DAPI (Beyotime, Haimen, China) antibodies.

### 2.6. Lentiviral shRNA Depletion and qRT-PCR

To overexpress or deplete FBXO11 and ZEB1, cells were infected with lentiviral vectors or shRNAs. After puromycin selection, RNA was extracted using Trizol (Thermo Fisher Scientific, Waltham, MA, USA, 15596026). cDNA was synthesized using Takara RR047A-3. cDNA (Takara RR047A-3) was synthesized, and gene expression levels were measured by real-time PCR and normalized to β-actin. Primer and shRNA sequences are reported in Supplementary Tables S1 and S2.

### 2.7. In Vitro Ubiquitination Assay

Wild-type Ub, Ub-K48, and Ub-K63 plasmids with HA tags were co-transfected with the relevant plasmids. After 18 h, transfected cells were combined with 10 μM proteasome inhibitor MG132 for 6 h. The experimental manipulations were continued according to immunoprecipitation and Western blot analysis with indicated with Flag and HA (Proteintech, #81290-1-RR) antibodies.

### 2.8. Protein Degradation Assays

HEK293T cells were seeded in 6-well plates for 24 h and transfected with 0.5 μg of indicated expression plasmids. When indicated, 0.2 μg GFP was used as an internal transfection control. After 16 h, transfected cells were treated with 10 μM MG132 or 20 μM Chloroquine (MedChemExpress, Monmouth Junction, NJ, USA, HY-17589) for 8 h and collected with ice-cold whole-cell extraction buffer (25mM β-Glycerophospholipids PH 7.3, 2 mM EGTA, 10 mM EDTA, 10 mM β-mercaptoethanol, 0.1 M NaCl, 1% Triton X-100, protease inhibitor cocktail) for Western blot analysis.

### 2.9. ZEB1 Half-Life Assay

HEK293T cells were seeded in 6-well plates for 24 h and transfected with 0.5 μg of indicated expression plasmids and 0.2 μg GFP as an internal transfection control. After 24 h, transfected cells were treated with 50 μg/mL cycloheximide (Aladdin, Shanghai, China, C112766) for 0, 2, 4, 6, 8, or 10 h and collected with ice-cold whole-cell extraction buffer for Western blot analysis.

### 2.10. Transwell Invasion Assay

Transwell chamber membranes (24-well; 8 μM, Costar, Costar, Corning, NY, USA, #3422) were coated with fibronectin (Sigma-Aldrich). Cells (5 × 10^4^) were plated in the top chamber and incubated at 37 °C overnight. Invaded cells were fixed and stained with 1% crystal violet (Solarbio, Beijing, China). Cell numbers were then counted under a microscope.

### 2.11. Wound-Healing Migration Assay

Cells were seeded in 6-well plates and grown to confluence. A wound was created by scraping with a 200 μL tip. Cells were washed with PBS and cultured in serum-free medium. Wound areas were photographed at 0 and 48 h, and the healing sizes were measured.

### 2.12. Mouse Subcutaneous Tumor Formation Assay

This study followed the Guide for the Care and Use of Laboratory Animals (Eighth Edition) and was approved by the Animal Ethics Committee of Changchun Wish Technology Company. After stable expression and screening of the corresponding plasmid cDNA in A549 cells infected with lentivirus, the cells were diluted to 5 × 10^6^. Cells were injected subcutaneously into the hindlimbs of 4–6-week-old nude mice (Balb/c-nu/nu). Tumor growth was observed and recorded. After growth to three weeks, the mice were sacrificed and dissected. Tumors were fixed with 4% PFA and paraffin-embedded, and 5 μM thick tumor sections were made. H&E staining was performed, and the sections were observed and photographed under a microscope.

### 2.13. Molecular Docking

We uploaded the FBXO11 protein sequence and the ZEB1 CZF domain (725-1125 amino acids) truncated protein sequence to LiHDOCK SERVER software (http://huanglab.phys.hust.edu.cn/, accessed on 13 October 2023) for protein–protein docking. Then, the docking combination with the best docking effect (model No. 1) was selected based on the docking score and confidence score and analyzed using PyMOL.

### 2.14. Kaplan–Meier Plot

We selected patient cohorts from the KM-PLOTTER website (http://kmplot.com/). We selected histological data from 1161 patients with lung adenocarcinoma.

### 2.15. Statistical Analysis

Patient sample data were downloaded from the TCGA database and grouped according to high and low FBXO11 expression. To investigate FBXO11 expression in the LUAD, we applied independent-sample *t*-tests to unpaired samples and paired *t*-tests to paired samples. Categorical data, including sex and tumor differentiation, were analyzed using chi-square tests. All analyses were two-sided and conducted with R 3.2.0 and SPSS 16.0.2. Statistical significance was set at *p* < 0.05.

## 3. Results

### 3.1. ZEB1 as the Major Transcription Factor Induces EMT in Lung Cancer Cells

**Figure 1:**
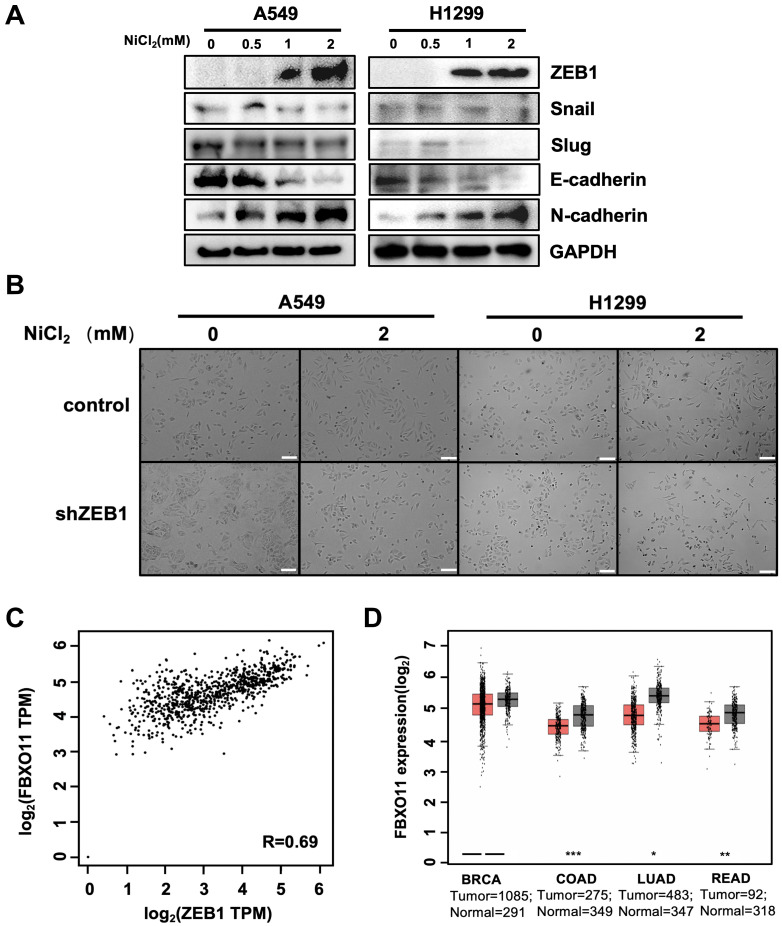
EMT is mainly induced in lung cancer cells by transcription factor ZEB1. (**A**) ZEB1 expression is increased in NiCl_2_-induced EMT. A549 and H1299 cells were treated with different concentrations of NiCl_2_ for 2 d. Immunoblotting assays were performed to detect the expression of each EMT marker. (**B**) Knockdown of ZEB1 in cells resistant to the induction effect of NiCl_2_. A549 and H1299 shNC or shZEB1 cells were treated with 2 mM NiCl_2_, and changes in cell morphology were observed under a microscope. Scale bar: 100 μm. (**C**) FBXO11 is positively correlated with ZEB1. Correlation analysis of LUAD data for TCGA and GTEx on the GEPIA website showed a correlation coefficient of R = 0.69 (*p* = 0; *p* < 0.01). TPM: transcripts per million. (**D**) FBXO11 exhibited higher expression in the normal tissue samples. The GEPIA database was used for FBXO11 expression in normal tissues and tumors (* *p* < 0.05, ** *p* < 0.01, *** *p* < 0.001).

To clarify the major types of transcription factors that induce EMT in lung cancer cells, we used nickel chloride (NiCl_2_) to induce EMT in human non-small cell lung cancer cell lines A549 and H1299. NiCl_2_ induces epithelial–mesenchymal transition (EMT) and enhances cellular invasiveness by generating reactive oxygen species (ROS) and altering DNA methylation, inhibiting the expression of E-cadherin and promoting the upregulation of N-cadherin and vimentin [32]. This mechanism has been validated in a variety of cell models, including lung cancer and renal tubular epithelial cells [33,34]. The expression of E-cadherin gradually decreased with increasing NiCl_2_ concentration, while ZEB1 expression was upregulated. It is noteworthy that the protein expression of Snail and Slug did not change (Figure 1A). The cell morphology shifted towards mesenchymal cell morphology with 2 mM NiCl_2_, while the knockdown of ZEB1 was able to resist the induction effect of NiCl_2_ (Figure 1B). Our previous study demonstrated that FBXO11 ubiquitinates and degrades Snail through the proteasome, thereby blocking Snail-induced EMT and inhibiting tumor metastasis in breast cancer [25,26]. Here, we speculated that FBXO11 might have a similar regulatory effect on ZEB1 in lung cancer cells. Using GEPIA, we performed gene correlation analysis using Transcripts Per Million (TPM) for lung cancer data in the TCGA/GTEx database and found that the ZEB1 gene was positively correlated with the FBXO11 gene (Figure 1C). To explore the role of FBXO11 in the development, progression, and prognosis of cancer, we investigated its expression in normal tissues and tumors in the TCGA and GETx databases (http://gepia.cancer-pku.cn) and found that FBXO11 was highly expressed in a variety of normal tissue compared to tumors, including BRCA, COAD, LUAD, and READ (Figure 1D).

We screened lung adenocarcinoma samples against FBXO11 expression in the TCGA clinical database. FBXO11 expression was not significantly correlated with the ages of the patients, sex, radiation therapy, or smoking history. It is noteworthy that the impact of FBXO11 expression on tumor grade is more likely to be seen in the early stages (I–II) of the tumor, when cancer cells are about to develop and have spread to a small extent (Table 1).

### 3.2. FBXO11 Associates with ZEB1

**Figure 2:**
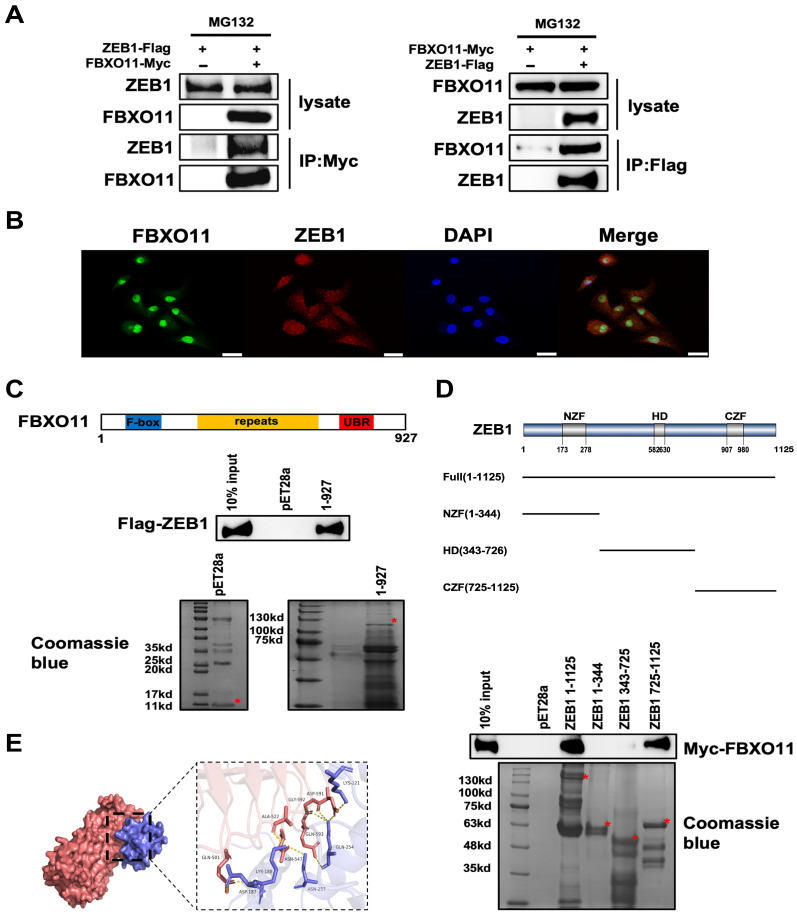
FBXO11 associates with ZEB1. (**A**) FBXO11 interacted with ZEB1 in a co-immunoprecipitation (co-IP) assay. HEK293T cells were transfected with Myc-tagged FBXO11 and Flag-tagged ZEB1 as indicated. Cell lysates were immunoprecipitated with either anti-Myc or anti-Flag antibodies and immunoblotted with anti-ZEB1 and anti-FBXO11 antibodies. (**B**) FBXO11 and ZEB1 co-location in the nucleus. Immunofluorescence assay probe colocalization of FBXO11 (green) and ZEB1 (red). Scale bar: 50 µm. (**C**) His pulldown assays showing FBXO11 bound to ZEB1 protein. A Coomassie blue staining image of PAGE gel is shown below to confirm the expression of pET28a and FBXO11. (**D**) His pulldown assays showing the CZF domain of ZEB1 bound to FBXO11 protein. A Coomassie blue staining image of PAGE gel is shown below to confirm the expression of various forms of ZEB1 proteins. (**E**) Molecular docking of FBXO11 protein and the ZEB1 CZF domain (725-1125 amino acids) truncated protein.

To investigate the interaction between FBXO11 and ZEB1, we first transfected the Myc-tagged FBXO11 and Flag-tagged ZEB1 expression constructs into HEK293T cells. Immunoprecipitation of Myc-tagged FBXO11 pulled down Flag-tagged ZEB1. In a reciprocal assay, immunoprecipitation of Flag-tagged ZEB1 also pulled down Myc-tagged FBXO11 (Figure 2A). An immunofluorescence assay further confirmed colocalization of FBXO11 and ZEB1 in HEK293T cells within the nucleus (Figure 2B). Next, we validated the protein interactions and mapped interacting domains by in vitro His pulldown assay. FBXO11 protein consists of an N-terminal F-box motif, a C-terminal zinc-finger-like UBR domain, and several repetitive cysteine-enriched domains located in the center [15]. The His pulldown assays showed the interaction between FBXO11 and ZEB1 (Figure 2C). ZEB1 consists of a central homeodomain (HD) and zinc-finger clusters at the N terminal (NZF) and C terminal (CZF) [10]. ZEB1 and its truncated mutants were expressed as His fusion protein. The ZEB1 C zinc-finger domain is indispensable for binding to FBXO11 by in vitro his pulldown assay (Figure 2D). After determining the binding domain, we simulated the interaction pattern of FBXO11 and ZEB1 C zinc finger using Z dock. The docking results show that the Z-dock docking fraction of FBXO11 and ZEB1 is 1742.563. Residues near the protein–protein interaction interface can form hydrogen bonds, which help stabilize the complex. PyMOL 2.3.0 was used to analyze the docking interaction patterns (Figure 2E). Overall, these results indicate that FBXO11 interacts with ZEB1 in vitro.

### 3.3. FBXO11 Stabilizes ZEB1 though Ubiquitination Acticity

**Figure 3:**
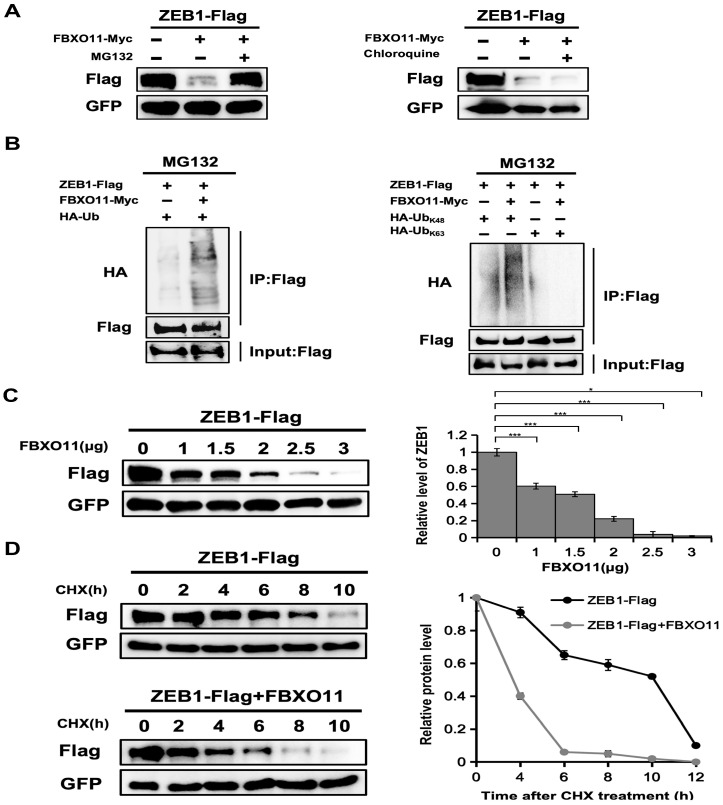
FBXO11 stabilizes ZEB1 though ubiquitination activity. (**A**) FBXO11 promotes ZEB1 proteasomal degradation. ZEB1-Flag was cotransfected with FBXO11-Myc in HEK293T cells, together with GFP, as well as an empty vector, and treated with DMSO, MG132, or chloroquine as indicated. The expressions of ZEB1 and GFP were assessed by Western blot. (**B**) FBXO11 promotes K48 polyubiquitinated chain generation of ZEB1 protein. In cellular ubiquitination assays, FBXO11-Myc was co-transfected with ZEB1-Flag plasmids or with HA-Ub-K63 and HA-Ub-K48 plasmids. Western blot was performed, and cell lysates were immunoprecipitated with anti-Flag antibody, then detected by anti-Ub antibody to poly-Ub levels. (**C**) FBXO11 degrades ZEB1 protein in a concentration-dependent manner. HEK293T cells were transfected with ZEB1-Flag, GFP, or FBXO11-Myc for 48 h, and cell lysates were immunoblotted and screened with anti-Flag antibody. Quantities were calculated as fold changes of 0 μg FBXO11. Error bars represent the SD from 3 independent experiments. * indicates *p* < 0.05, and *** indicates *p* < 0.001. Each experiment was repeated at least three times. (**D**) Exogenous FBXO11 accelerates ZEB1 protein turnover. HEK293T cells were transfected with ZEB1-Flag and GFP, in combination with FBXO11-Myc, and treated with cycloheximide (CHX) as indicated. Cell lysates were subjected to western blot analysis with anti-ZEB1 and anti-GFP antibodies. The graph shows the quantification of ZEB1 protein levels (based on the band intensity from the gels) normalized to GFP over the time course. The ZEB1 protein level at the 0 h time point of CHX treatment was set as 100%. The experiment was repeated three times, and a representative experiment is presented.

Given that FBXO11 is an E3 ubiquitin ligase, we investigated its role in ZEB1 ubiquitination and degradation. Co-transfection with FBXO11 led to ZEB1 degradation, which was blocked by proteasome inhibitor MG132 but not by chloroquine, demonstrating that overexpression of FBXO11 caused the proteasomal degradation of ZEB1 (Figure 3A). FBXO11 overexpression significantly increased ZEB1 ubiquitination, particularly K48-linked ubiquitination (Figure 3B). As FBXO11 levels increased, ZEB1 protein levels decreased, indicating concentration-dependent degradation by FBXO11 (Figure 3C). FBXO11 also accelerated ZEB1 turnover but not that of GFP (Figure 3D). Taken together, the results suggest that FBXO11 promotes polyubiquitination and the degradation of ZEB1 protein.

### 3.4. FBXO11 Regulates the Expression of EMT-Related Factors

**Figure 4:**
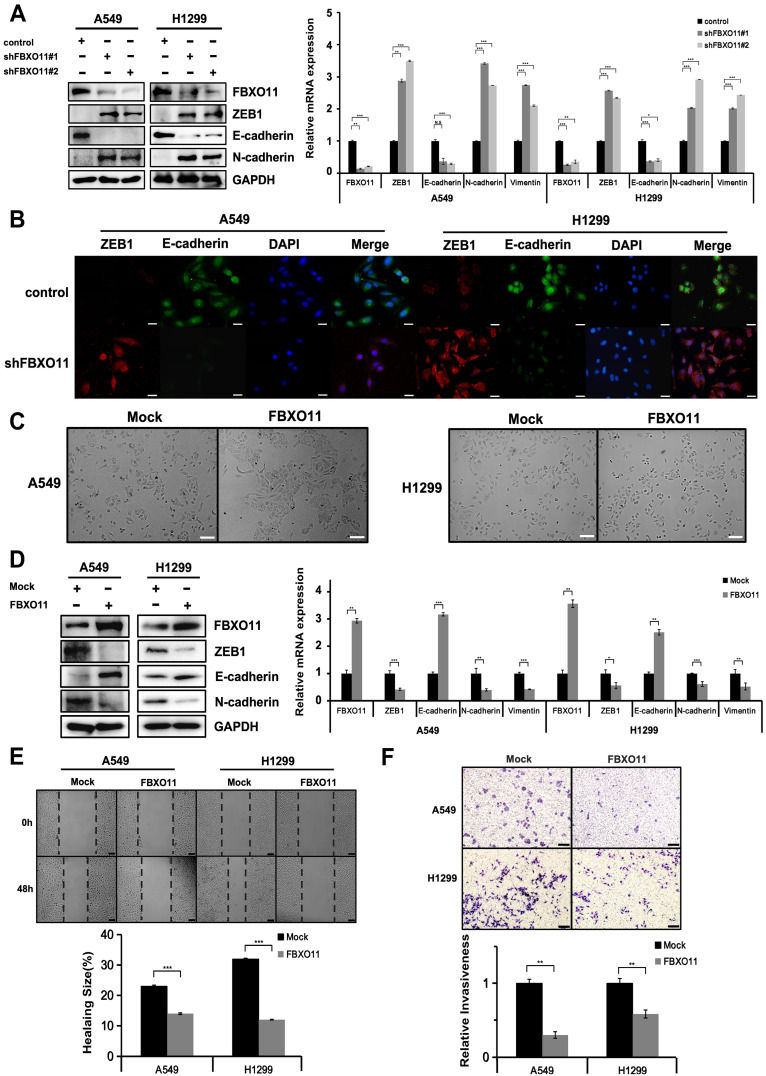
FBXO11 affects the expression of EMT-related factors. (**A**) Endogenous FBXO11 knockdown changes the expression of ZEB1 and EMT marker genes in lung adenocarcinoma cells. Immunoblotting analysis and quantitative RT-PCR analysis of ZEB1 and EMT markers in A549 and H1299 cells was transduced with lentiviral shRNAs targeting control or FBXO11. Error bars represent the SD from 3 independent experiments. * indicates *p* < 0.05, ** indicates *p* < 0.01, and *** indicates *p* < 0.001. (**B**) Immunofluorescence analysis of ZEB1 and E-cadherin protein expression in control and shFBXO11 of A549 and H1299 cells (ZEB1, red; E-cadherin, green; DAPI, blue). Scale bar: 50 μm. (**C**) FBXO11 overexpression affects changes cells into the mesenchymal phenotype. Cell morphology in A549 and H1299 cells with overexpressed Mock or FBXO11. Scale bars: 100 μm. (**D**) The overexpression of FBXO11 alters the expression of ZEB1 and EMT marker genes in lung adenocarcinoma cells. Immunoblotting and quantitative RT-PCR analysis of ZEB1 and EMT markers in A549 and H1299 cells transduced with Mock or FBXO11. Error bars represent the SD from 3 independent experiments. ** indicates *p* < 0.01, and *** indicates *p* < 0.001. (**E**) Wound-healing assay showing the migration of A549 and H1299 cells after transfection with Mock or FBXO11-Myc. Representative images are shown 0 and 48 h post scratch (*n* = 3). Scale bars: 100 μm. (**F**) Transwell assay showing the invasion of A549 and H1299 cells transfected with Mock or FBXO11-Myc (*n* = 3). Scale bars: 100 μm.

To explore the role of FBXO11 in the modulation of the migration and invasion of lung cancer cells, we examined the expression of genes that regulate EMT in A549 and H1299 cells. The depletion of FBXO11 caused marked upregulation of ZEB1 protein levels and downregulation of epithelial marker E-cadherin. Mesenchymal marker N-cadherin was increased (Figure 4A). The conversion of E-cadherin and N-cadherin expression is one of the hallmarks of EMT. Similar results were observed in H1299 lung cancer cells (Figure 4A). Immunostaining also showed that FBXO11 depletion increased the expression of ZEB1 and decreased E-cadherin (Figure 4B). When A549 and H1299 cells were transduced with lentivirus-expressing FBXO11, endogenous ZEB1 and N-cadherin protein and mRNA levels decreased, while E-cadherin protein and mRNA expression increased (Figure 4D). The upregulation of E-cadherin is indicative of EMT and is expected to decrease cell motility and invasion. As expected, the overexpression of FBXO11 in A549 and H1299 cells strengthened intercellular adhesion, resulting in a more clustered morphology (Figure 4C). Concomitantly, in a wound-healing migration assay (Figure 4E) and transwell invasion assay (Figure 4F), the migratory and invasive abilities of the cells were weakened. These results suggest that FBXO11 can regulate the expression of EMT-related factors and affect the migration and invasion in lung cancer cells.

### 3.5. The FBXO11–ZEB1 Axis Regulates the EMT Pathway in LUAD

**Figure 5:**
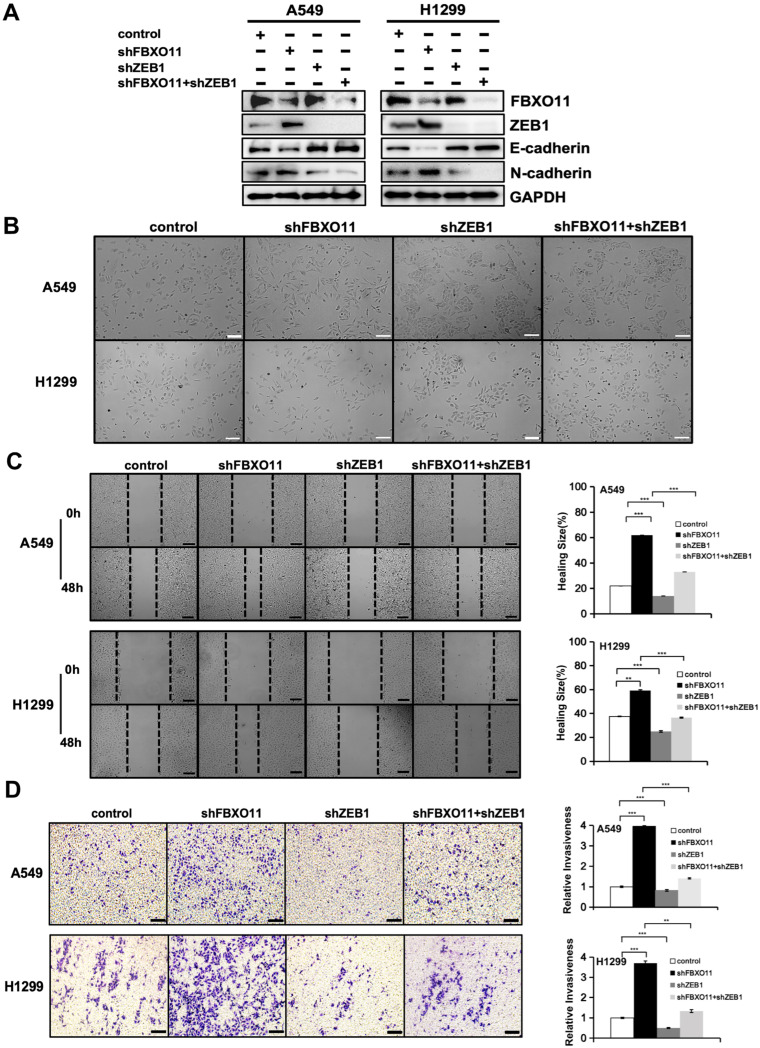
The FBXO11–ZEB1 axis regulates the EMT pathway in LUAD. (**A**) Detection of FBXO11 or ZEB1 in A549 and H1299 cells expressing the indicated shRNAs. (**B**) The FBXO11–ZEB1 axis regulates epithelial–mesenchymal cell morphology in lung cancer cells. Morphological changes in A549 and H1299 control, shFBXO11, and shZEB1 and co-knockdown of ZEB1 and FBXO11 cells. Scale bars: 100 μm. (**C**) FBXO11 affects the migratory ability of A549 and H1299 cells via ZEB1. Scratching experiments were performed to analyze changes in the migratory capacity of A549 and H1299 control, shFBXO11, and shZEB1 and co-knockdown of ZEB1 and FBXO11 cells. Scale bar: 100 μm. The area of cell invasion was counted (*n* = 6). ** indicates *p* < 0.01, *** indicates *p* < 0.001. (**D**) Cell invasiveness is moderated in vitro via the FBXO11–ZEB1 axis. A transwell assay to was conducted to analyze changes in the invasive capacity of A549 and H1299 control, shFBXO11, and shZEB1 and co-knockdown of ZEB1 and FBXO11 cells. Scale bar: 100 μm. The count of cells crossing the basement membrane was *n* = 6. ** indicates *p* < 0.01, and *** indicates *p* < 0.001.

Since ZEB1 is a core regulator of EMT, we investigated whether FBXO11 depletion enhanced EMT through the increased expression of ZEB1. Compared with FBXO11 knockdown alone, FBXO11 and ZEB1 double knockdown increased E-cadherin and decreased N-cadherin protein expression, which is similar to the result of ZEB1 knockdown alone (Figure 5A). The depletion of FBXO11 in A549 and H1299 cells gave rise to a predominance of dispersed and elongated single cells (Figure 5B). Similar to ZEB1 knockdown alone, FBXO11 and ZEB1 double knockdown cells showed tight connections and regular arrangement (Figure 5B). Consistent with protein expression and cell morphology, FBXO11 and ZEB1 double knockdown caused decreased cell mobility (Figure 5C) and aggressiveness (Figure 5D). Moreover, ZEB1 knockdown alone showed the lowest cellular mobility and aggressiveness. Together, these results demonstrate that FBXO11 regulated the migration and invasion of A549 cells through ZEB1.

### 3.6. FBXO11 Inhibits LUAD Progression by Stabilizing ZEB1

**Figure 6:**
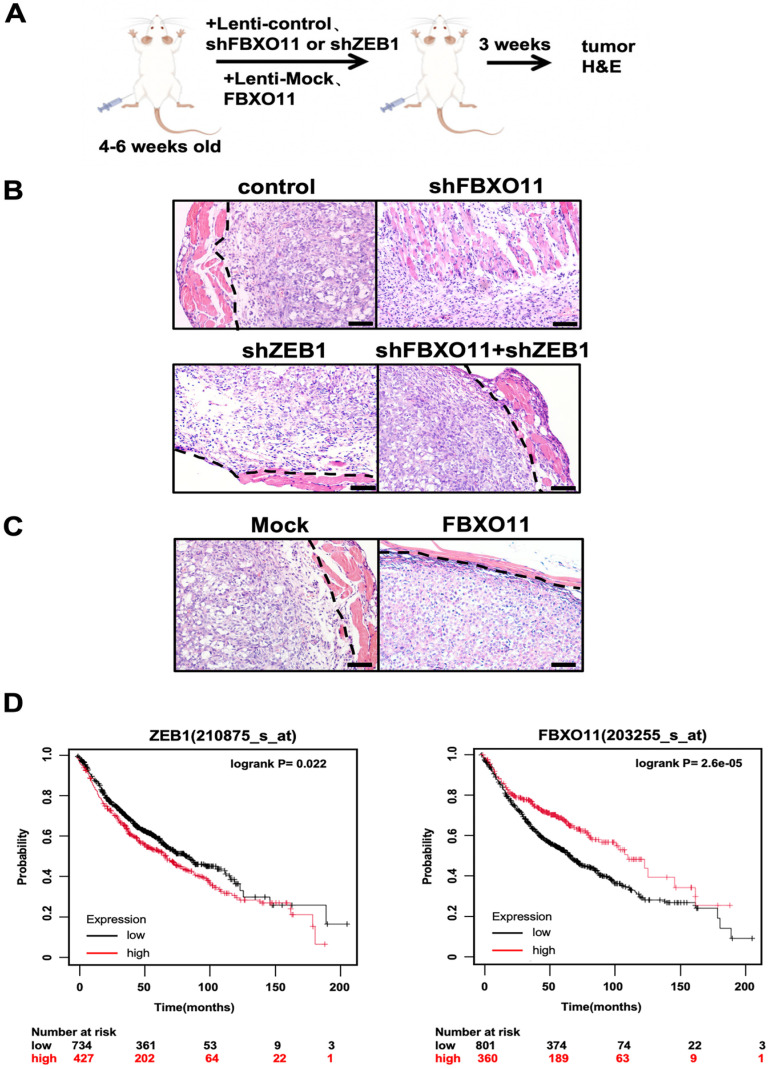
FBXO11 inhibits LUAD progression by stabilizing ZEB1. (**A**) Diagram of mouse hindlimb injection with A549 cells. (**B**) The FBXO11–ZEB1 axis controls tumor cell infiltration. Xenograft experiments were performed to analyze changes in the invasive ability of control, shFBXO11, shZEB1, co-knockdown, ZEB1, and FBXO11 cells in mice. Scale bars: 50 μm. (**C**) Xenograft experiments were performed to analyze changes in the invasive ability of A549, Mock, and FBXO11 cells in mice. Scale bars: 50 μm. (**D**) High ZEB1 and low FBXO11 expression predict poor prognosis. Kaplan–Meier plot of overall survival of lung cancer patients stratified by the expression of ZEB1 and FBXO11 genes. Data were obtained from KMplot.com.

**Figure 7:**
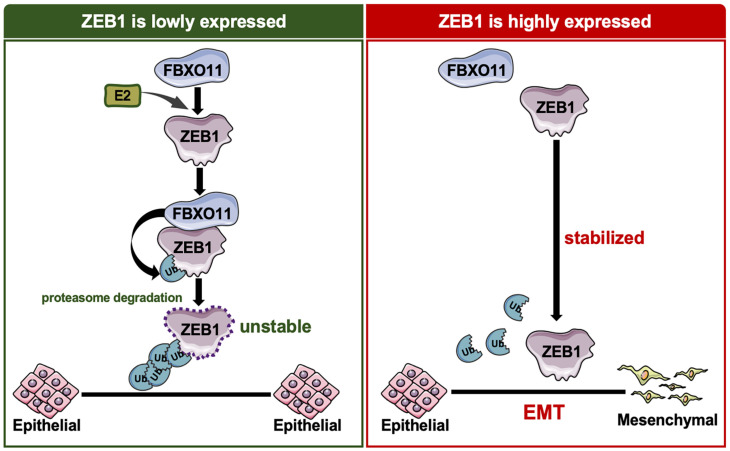
FBXO11 regulates EMT and metastasis through ZEB1 in LUAD. FBXO11-dependent ubiquitination and ZEB1 protein stability regulate the invasion and metastasis of lung cancer cells.

To explore the physiological role of FBXO11 and ZEB1 in vivo, we transplanted A549 cells into nude mice (Balb/c-nu/nu) with an immunodeficient system suitable for tumor transplantation and immunology research [35] (Figure 6A). Tumors with FBXO11 deletion showed significant local invasion, while the results for tumors with FBXO11 and ZEB1 double knockdown were similar to those of tumors with ZEB1 single knockdown, with clear tumor boundaries (Figure 6B). Xenograft tumors derived from control A549 cells and FBXO11 overexpression cells both formed well-defined envelope tumors (Figure 6C). These results show that FBXO11 can affect the invasion and metastasis of lung cancer cells by regulating ZEB1 expression both in vivo and in vitro. In the lung cancer database organized by Kaplan–Meier plot, analysis demonstrated that ZEB1 expression levels were negatively correlated with patient survival, and patients with high levels of FBXO11 expression in their tumors had higher overall survival rates and survived longer than patients with low levels of FBXO11 expression (Figure 6D). This is in line with the trend of our findings. Taken together, we confirm that EMT transcription factor ZEB1 plays a major role in the invasion and metastasis of lung cancer and that FBXO11 can ubiquitinate ZEB1 and be recognized by the proteasome for degradation, thereby inhibiting the invasion and metastasis of lung cancer cells (Figure 7).

## 4. Discussion

Lung cancer is the leading cause of cancer death and has one of the lowest 5-year survival rates among all cancer types [36,37]. It is often accompanied by metastases in late stages [38,39]. During tumorigenesis, progression, and metastasis, cancer cells invade the stroma, enter the bloodstream, and colonize distant organs. It is well-established that epithelial–mesenchymal transition (EMT) and mesenchymal–epithelial transition (MET) are critical for cancer cell dissemination [40]. Classical EMT transcription factors like Snail, Slug, Twist, and ZEB1/2 are upregulated in cancer cells, which enhances their invasive and metastatic potential [41]. ZEB1, in particular, is highly expressed in various cancers and plays a crucial role in tumor progression and metastasis. ZEB1 interacts with the tumor microenvironment, including immune cells, and exhibits varying degrees of radioresistance and drug resistance [42,43]. The SCF (Skp1-Cul1-F-box) ubiquitin ligase complex is the largest family of E3 ubiquitin ligases that mediate the ubiquitination of post-translationally modified target proteins or substrates and their degradation by the proteasome [23,44,45]. F-box proteins are a large family of proteins, of which FBXO11 is a member, and act as an E3 ligase that can play a role in carcinogenesis by regulating oncogenes or tumor suppressors [46,47,48]. 

This study systematically investigated the molecular mechanisms through which E3 ubiquitin ligase FBXO11 mediates the ubiquitination and degradation of ZEB1, thereby regulating the invasive metastasis of lung cancer cells. We demonstrated that the expression of the FBXO11–ZEB1 axis is strongly associated with the incidence and prognosis of non-small cell lung cancer (NSCLC), the most common type of lung cancer, and has potential as a therapeutic target. Specifically, EMT was induced in human NSCLC A549 cells by NiCl_2_, leading to the upregulation of ZEB1, while other EMT drivers remained unchanged, underscoring ZEB1’s primary role in the EMT pathway in lung adenocarcinoma cells. Our findings reveal that ZEB1 interacts with FBXO11, which enhances the polyubiquitination and proteasomal degradation of ZEB1 and reduces the intracellular half-life of ZEB1. Low FBXO11 expression in lung adenocarcinoma cells increases ZEB1 protein levels, decreases epithelial marker E-cadherin, and increases mesenchymal markers N-cadherin and vimentin, promoting EMT and enhancing cell migration and invasion. The overexpression of FBXO11 reduces ZEBs1 protein levels and maintains epithelial cell status, suggesting a better clinical prognosis. FBXO11 was also found to inhibit lung cancer cell migration and invasion in vivo in xenograft mice.

## 5. Conclusions

Our findings elucidate the roles played by FBXO11 and ZEB1 in lung adenocarcinoma from a fundamental molecular perspective. Future research should focus on elucidating the role of the FBXO11–ZEB1 axis in vivo, particularly in human lung cancer. This includes the use of animal models to study resistance and evaluate the therapeutic potential and off-target effects. Such a comprehensive could further validate our findings and explore the clinical implications of the interaction between FBXO11 and ZEB1. In addition, identifying small-molecule inhibitors of FBXO11 and studying their efficacy in preclinical models could pave the way for novel targeted therapies. Our ultimate goal is to develop effective therapies that improve survival and reduce mortality in lung cancer patients.

## Figures and Tables

**Table 1:** Correlation of FBXO11 expression with clinicopathological covariates in lung cancer patients.

Characteristic	FBXO11—High (*n* = 286)	FBXO11—Low (*n* = 286)	*p* Value *
Age (years), mean (SD)	66 (60.73)	66 (57.72)	0.407 †
Sex (n(%))			0.737
Women	153 (53.5)	157 (54.9)	
Men	133 (46.5)	129 (45.1)	
Radiation therapy			0.385
No	224 (78.4)	195 (68.2)	
YES	33 (11.5)	36 (12.6)	
Missing	29 (10.1)	55 (19.2)	
Smoking history			0.718
≤2	100 (35)	108 (37.8)	
>2	177 (61.9)	167 (58.4)	
Missing	9 (3.1)	11 (3.8)	
Tumor stage			0.146 ‡
i	169 (59.1)	141 (49.4)	
ii	56 (19.6)	77 (26.9)	
iii	47 (16.4)	46 (16.1)	
iv	12 (4.2)	15 (5.2)	
Missing	2 (0.7)	7 (2.4)	

* χ2 test or Fisher’s exact test. † Student’s *t* test. ‡ Mann–Whitney U test (non-parametric). Missing values are excluded for all statistical tests.

## Data Availability

The datasets used and analyzed in this paper are available from the corresponding author upon reasonable request.

## Supplementary Materials

The following supporting information can be downloaded at https://www.mdpi.com/article/10.3390/cancers16193269/s1: Figure S1: Morphological changes in A549 cells were induced by NiCl_2_; Figure S2: FBXO11 and UBR5 are not colocalized in the nucleus. Immunofluorescence assay probing the colocalization of FBXO11 (Green) and UBR5 (Red). Scale bar: 50 µm. Table S1: q-PCR primers; Table S2: Lentiviral packaging sequence; Original blot data file.
