# Supplementary material for: FBXO11 Mediates Ubiquitination of ZEB1 and Modulates Epithelial-to-Mesenchymal Transition in Lung Cancer Cells
Source: Cancers (Basel). 2024 Sep 26;16(19):3269. doi: 10.3390/cancers16193269 (PMC11476264; doi:10.3390/cancers16193269)
Supplement: Supplementary file 1 [file cancers-16-03269-s001.zip › manuscript-Supplementary Materials.pdf]

Figure S1

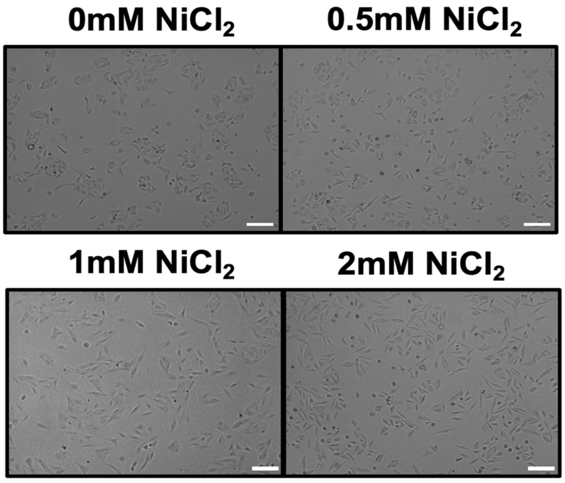

Figure S1. The morphological changes of A549 cells were induced by $NiCl_2$. A549 cells were treated with different concentrations of $NiCl_2$ for 2 d and the changes in cell morphology were observed under a microscope. Scale bar: 100 μm.

Figure S2

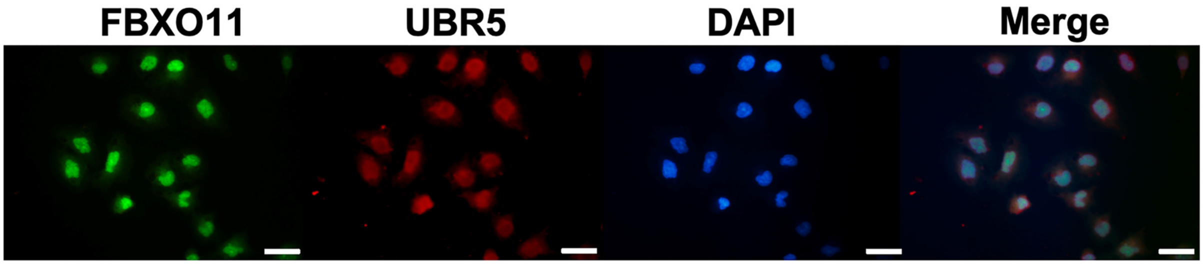

Figure S2. FBXO11 and UBR5 are not co-localised in the nucleus. Immunofluorescence assay probe colocalization of FBXO11 (Green), and UBR5 (Red). Scale bar: 50 µm.

Supplementary Table S1

q-PCR primers

Primers	Sequence
FBXO11：	Forward: TGGACGTGATGTTGGTGTGTTCACA
	Reverse: TCCTCCAGTCTGCCCATGGTGA
ZEB1：	Forward: GCCAATAAGCAAACGATTCTG
	Reverse: TTTGGCTGGATCACTTTCAAG
CDH1:	Forward: CAGTGAACAACGATGGCATT
	Reverse: CTGGGCAGTGTAGGATGTGA
CDH2:	Forward: TCAGGCTCCAAGCACCCCTTCA
	Reverse: ATGACGGCCGTGGCTGTGTT
VIM :	Forward: ATTGAGATTGCCACCTACAG
	Reverse: ATCCAGATTAGTTTCCCTCAG
β-actin：	Forward: AGAAAATCTGGCACCACACC
	Reverse: AGAGGCGTACAGGGATAGCA

Supplementary Table S2

Lentiviral packaging sequence

shRNA seq	
shFBXO11#1	GCTCACGGACAACACTTACAA
shFBXO11#2	TCAGGCCAGGAAGTCATGTTT
shZEB1#1	CCTCTCTGAAAGAACACATTA
shZEB1#2	GCTGTTGTTCTGCCAACAGTT
