# Supplementary material for: FBXO11 Mediates Ubiquitination of ZEB1 and Modulates Epithelial-to-Mesenchymal Transition in Lung Cancer Cells
Source: Cancers (Basel). 2024 Sep 26;16(19):3269. doi: 10.3390/cancers16193269 (PMC11476264; doi:10.3390/cancers16193269)

Raw data of immunoblot from Figure 1A

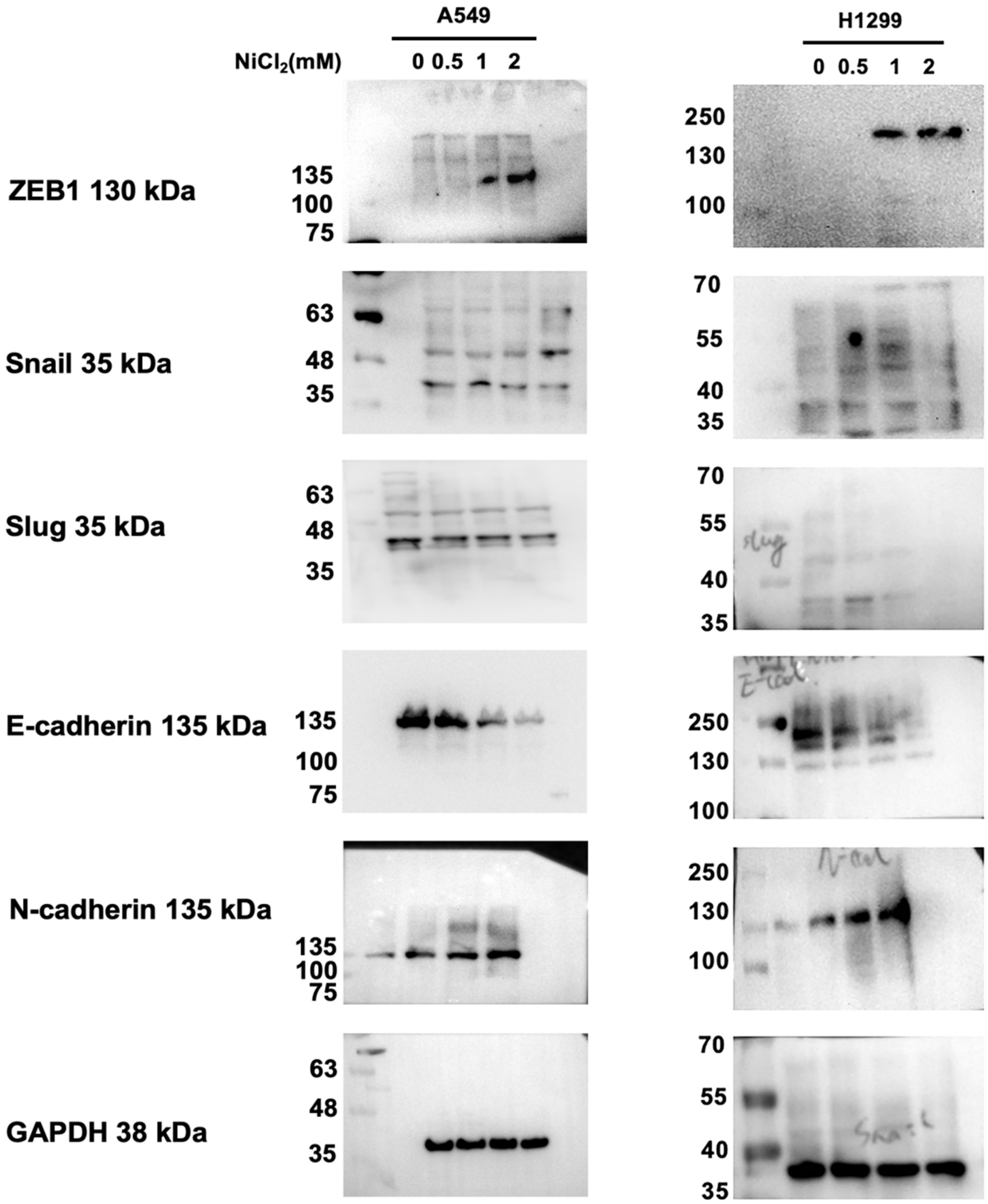

Raw data of immunoblot from Figure 2A

IP-Myc

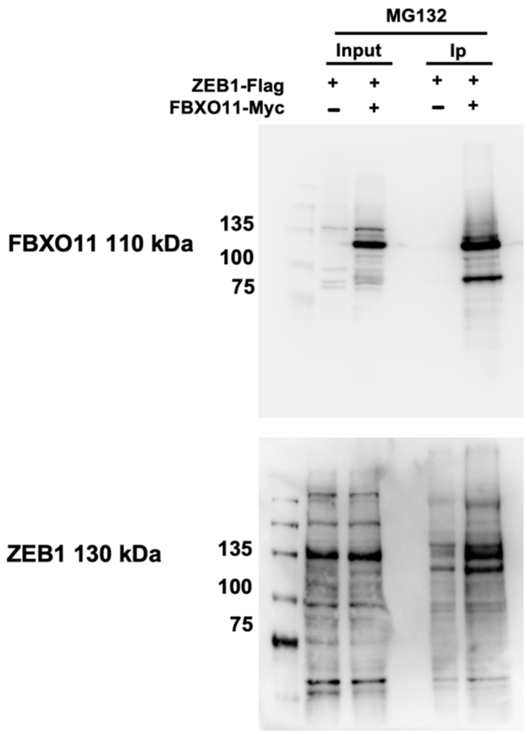

IP-Flag

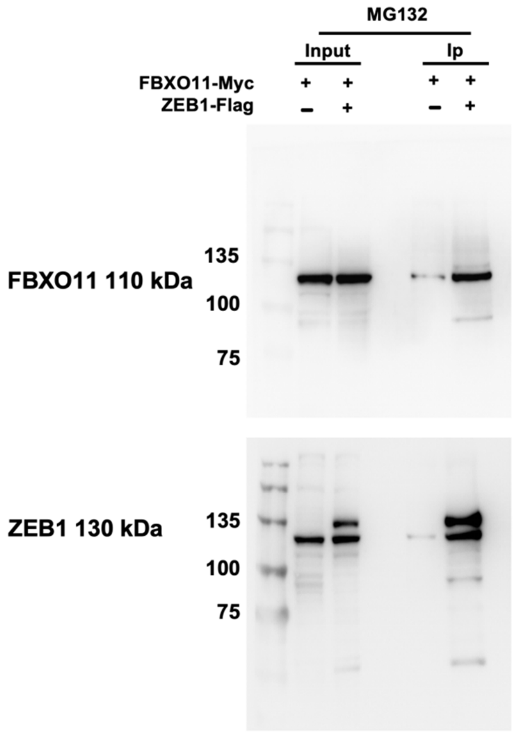

Raw data of immunoblot from Figure 2C

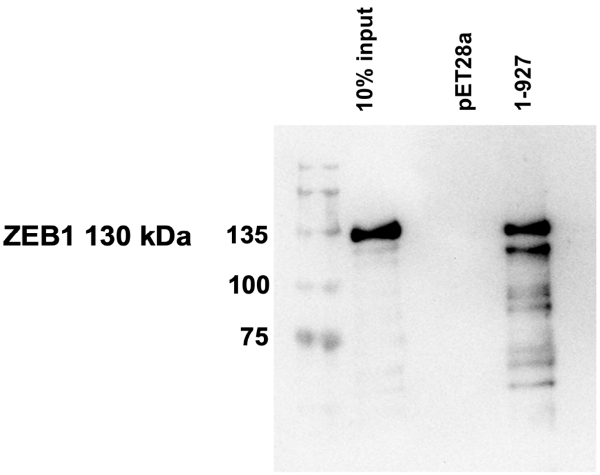

Raw data of immunoblot from Figure 2D

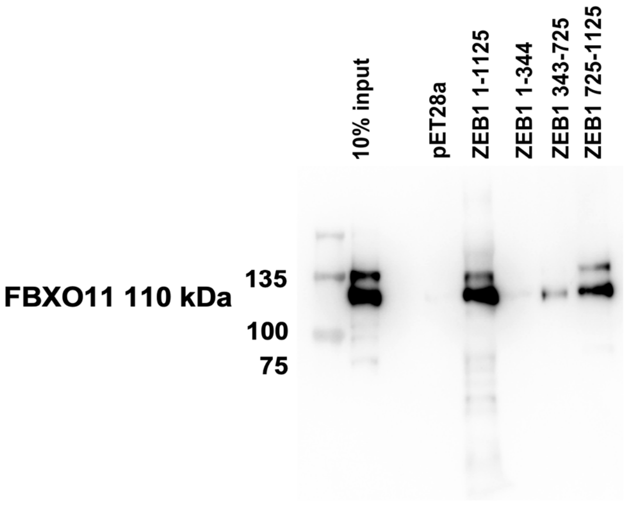

Raw data of immunoblot from Figure 3A

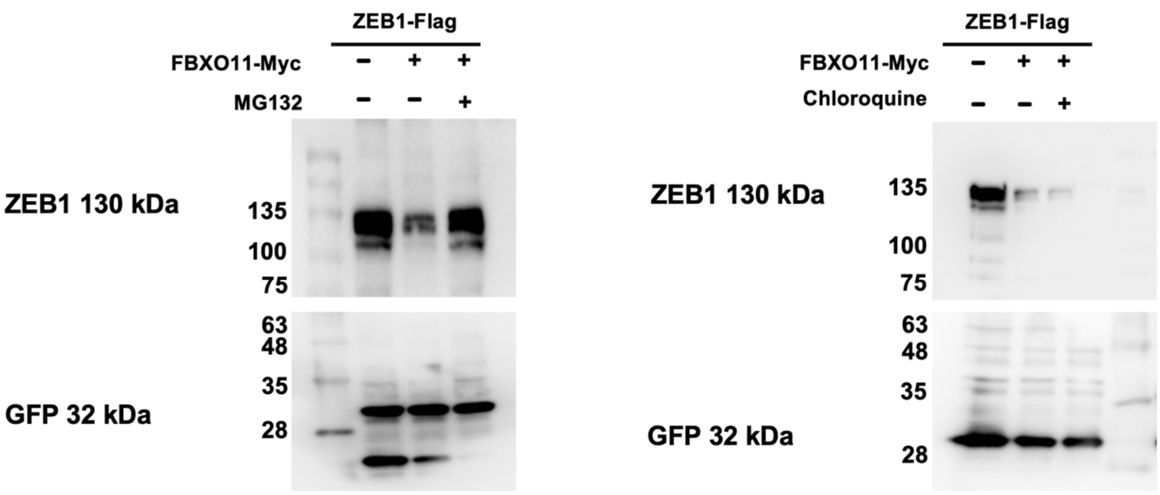

Raw data of immunoblot from Figure 3B

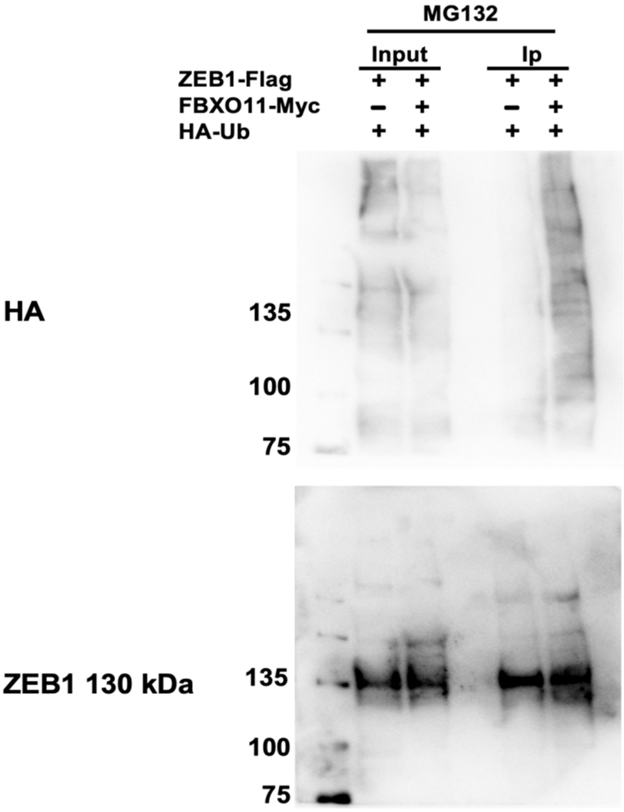

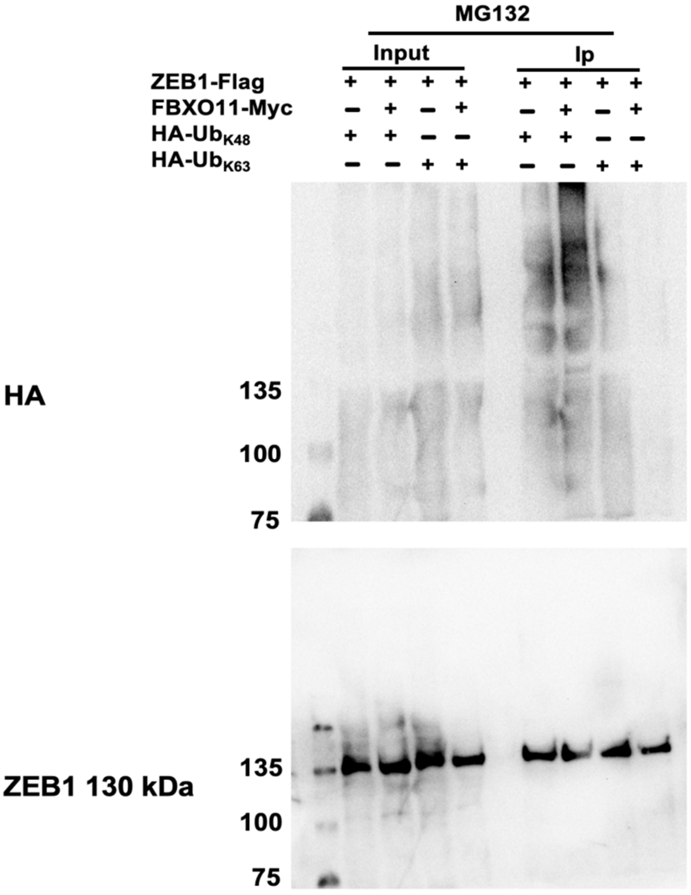

Raw data of immunoblot from Figure 3C

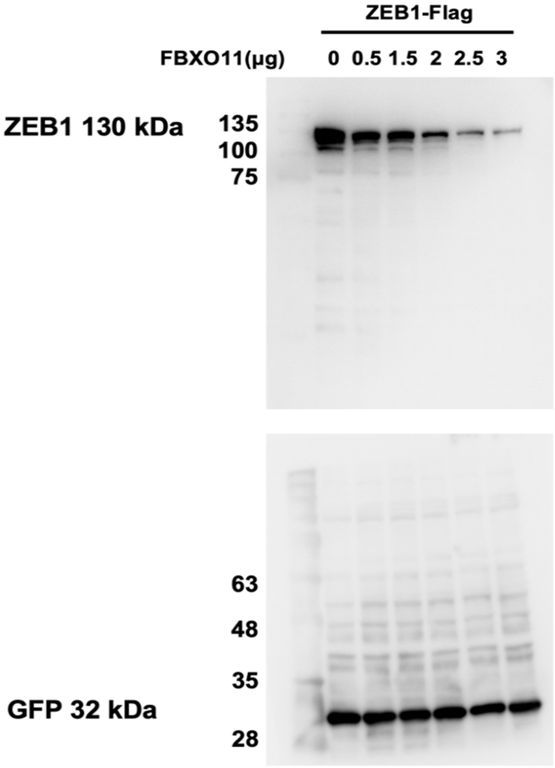

Raw data of immunoblot from Figure 3D

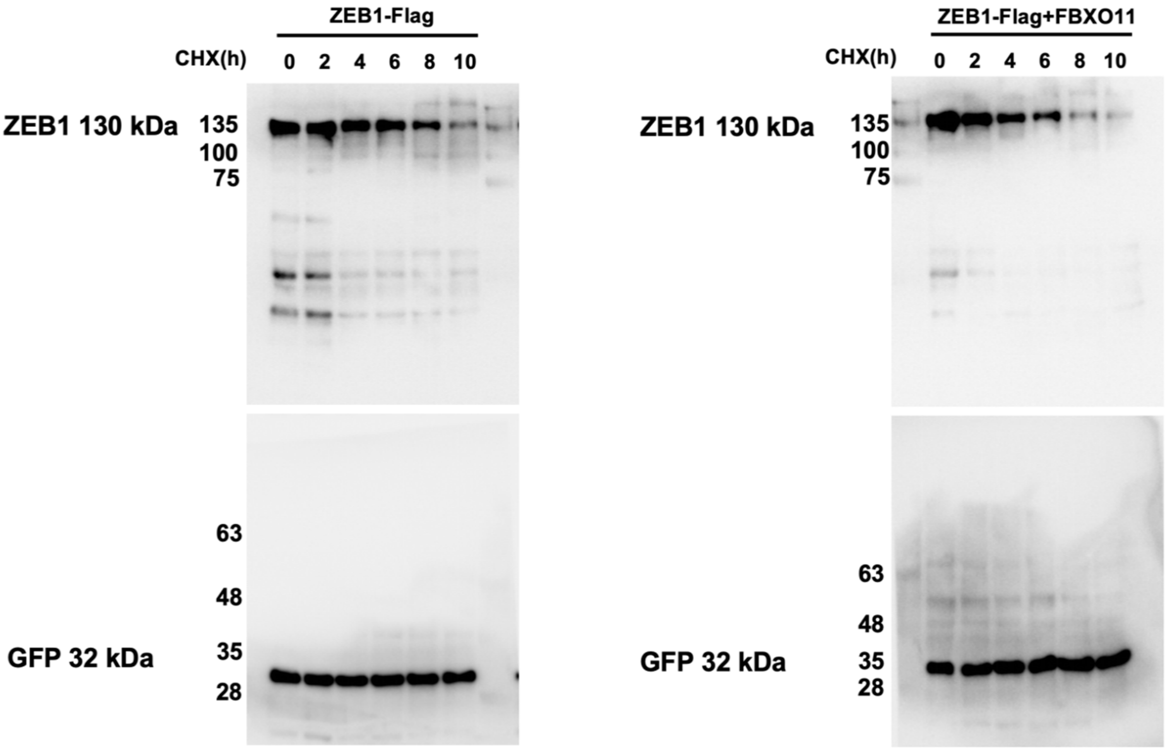

Raw data of immunoblot from Figure 4A

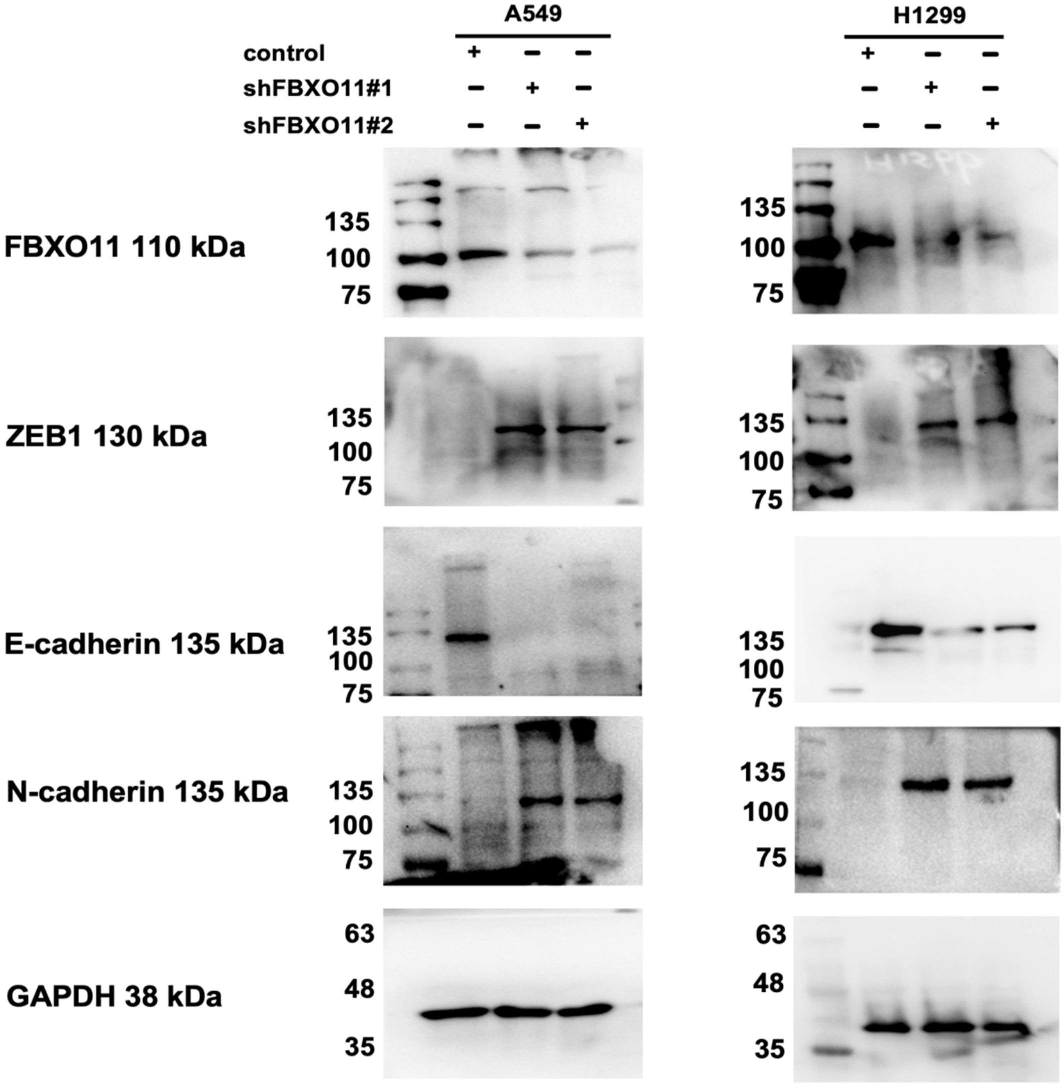

Raw data of immunoblot from Figure 4D

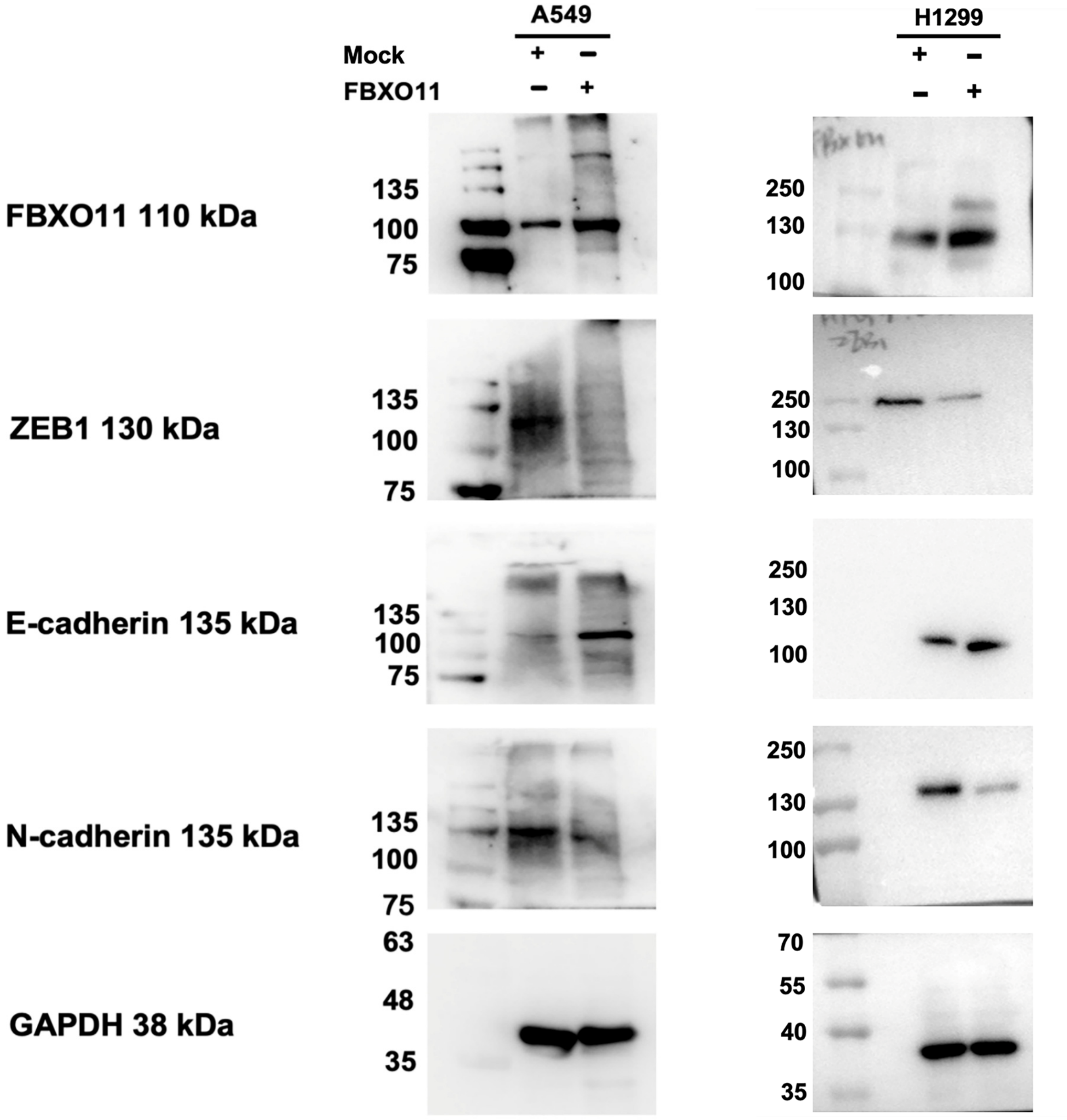

Raw data of immunoblot from Figure 5A

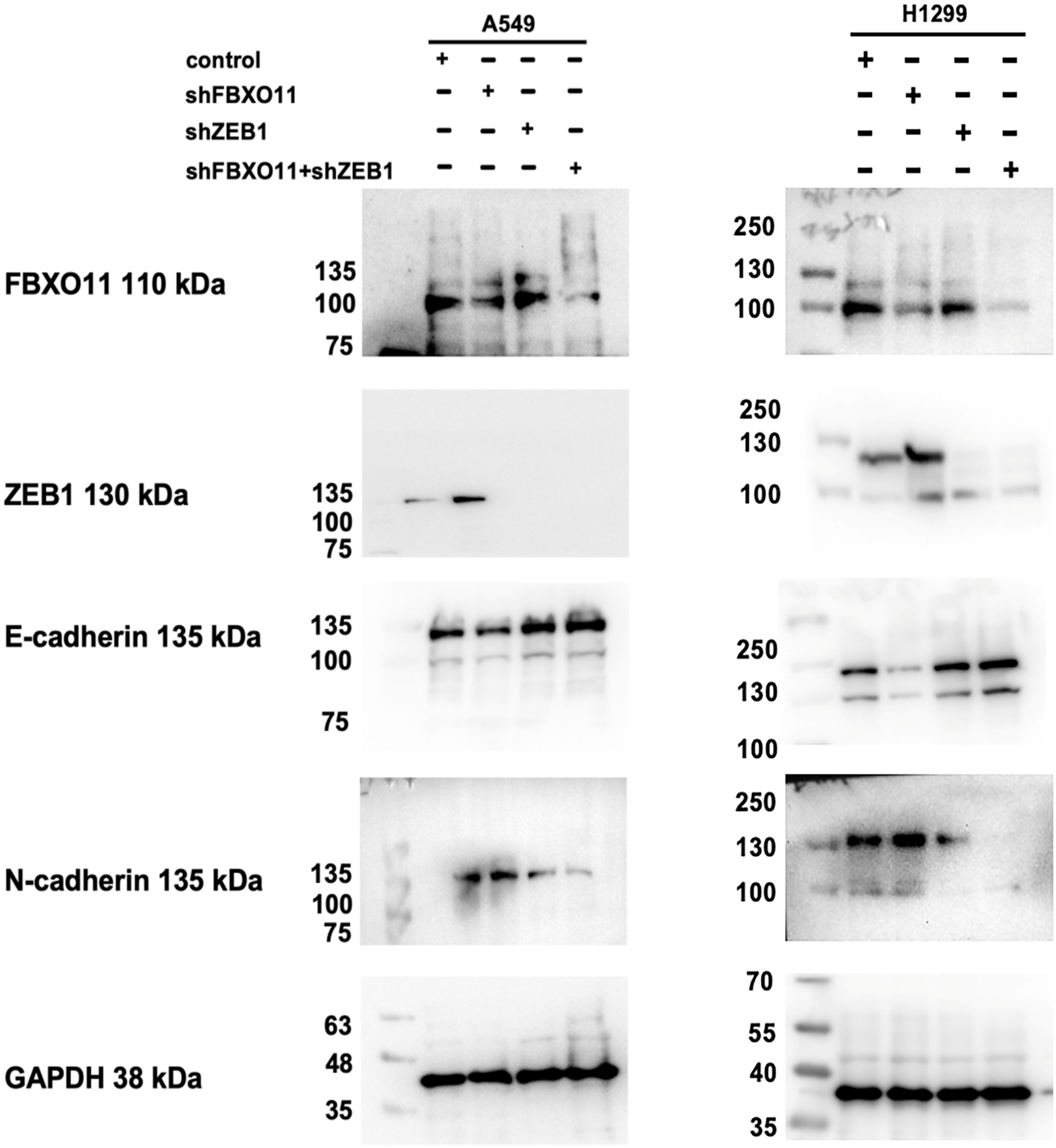

Supplement: Supplementary file 1 [file cancers-16-03269-s001.zip › original blot data.pdf]
